# Supplementary material for: ZmADF5, a Maize Actin-Depolymerizing Factor Conferring Enhanced Drought Tolerance in Maize
Source: Plants (Basel). 2024 Feb 24;13(5):619. doi: 10.3390/plants13050619 (PMC10935123; doi:10.3390/plants13050619)
Supplement: Supplementary file 1 [file plants-13-00619-s001.zip › Supplementary figures.pdf]

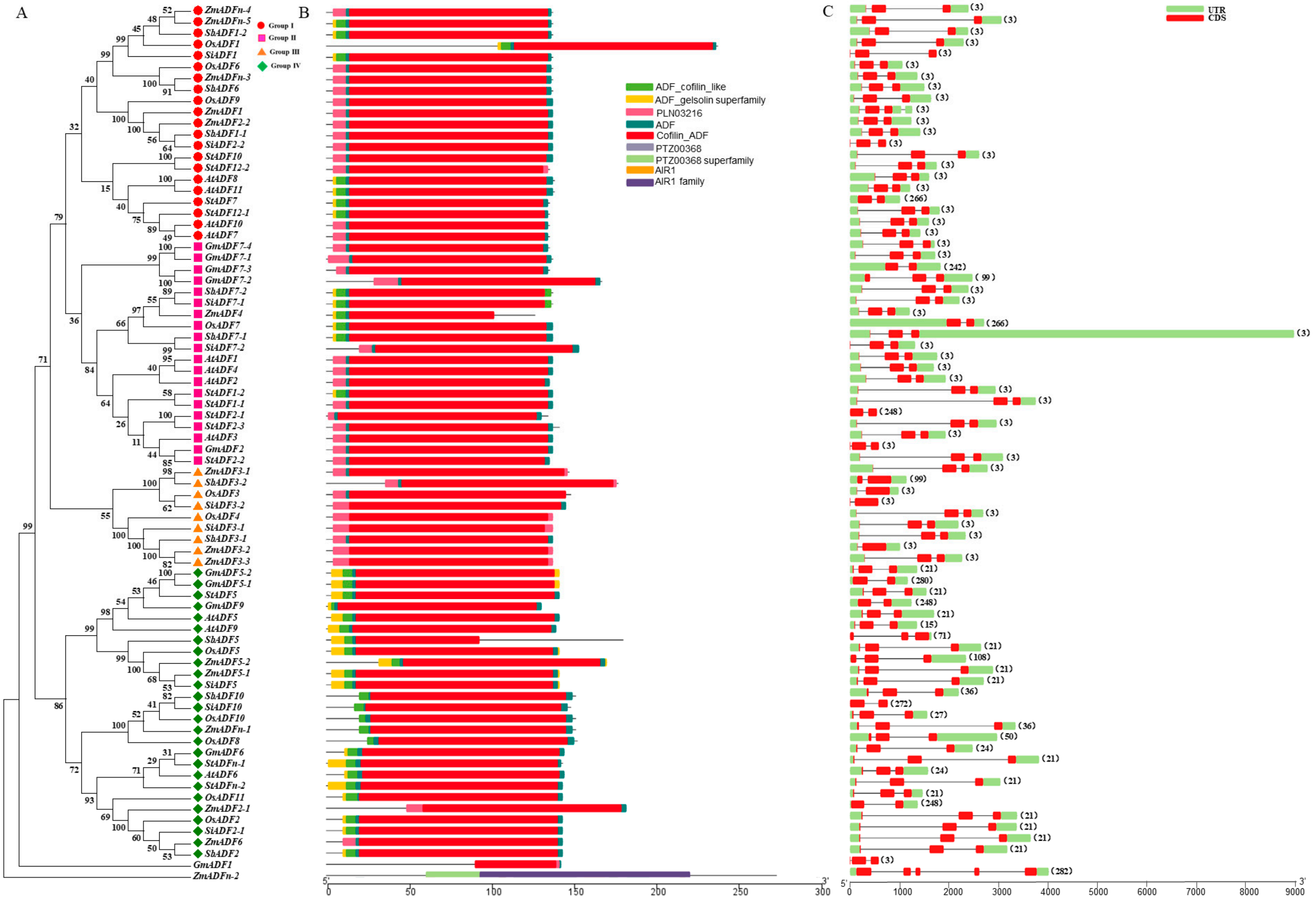
A
B
C
Group I
Group II
Group III
Group IV
ZmADFn-4
ZmADFn-5
SbADF1-2
OsADF1
SiADF1
OsADF6
ZmADFn-3
SbADF6
OsADF9
ZmADF1
ZmADF2-2
SbADF1-1
SiADF2-2
StADF10
StADF12-2
AtADF8
AtADF11
StADF7
StADF12-1
AtADF10
AtADF7
GmADF7-4
GmADF7-1
GmADF7-3
GmADF7-2
SbADF7-2
SiADF7-1
ZmADF4
OsADF7
SbADF7-1
SiADF7-2
AtADF1
AtADF4
AtADF2
StADF1-2
StADF1-1
StADF2-1
StADF2-3
AtADF3
GmADF2
StADF2-2
ZmADF3-1
SbADF3-2
OsADF3
SiADF3-2
OsADF4
SiADF3-1
SbADF3-1
ZmADF3-2
ZmADF3-3
GmADF5-2
GmADF5-1
StADF5
GmADF9
AtADF5
AtADF9
SbADF5
OsADF5
ZmADF5-2
ZmADF5-1
SiADF5
SbADF10
SiADF10
OsADF10
ZmADFn-1
OsADF8
GmADF6
StADFn-1
AtADF6
StADFn-2
OsADF11
ZmADF2-1
OsADF2
SiADF2-1
ZmADF6
SbADF2
GmADF1
ZmADFn-2
ADF_cofilin_like
ADF_gelsolin superfamily
PLN03216
ADF
Cofilin_ADF
PTZ00368
PTZ00368 superfamily
AIR1
AIR1 family
UTR
CDS
5'
3'
0
50
100
150
200
250
300
1000
2000
3000
4000
5000
6000
7000
8000
9000

Supplementary Figure S1 Phylogenetic analysis, conserved motifs, and gene structural analysis of all ADF genes. **(A)** Phylogenetic analysis of ADF genes in maize and six other plant species. At, *Arabidopsis thaliana*; Gm, *Glycine max*; Os, *Oryza sativa*; Sb, *Sorghum bicolor*; Si, *Setaria italica*; St, *Solanum tuberosum*; and Zm, *Zea mays*. **(B)** Conserved motif analysis of all ADF genes. The 20 motifs are represented by different colored boxes. **(C)** Structural diagram of all ADF genes. Numbers in parentheses after the structure diagram represent the length of the first exon of the ADF gene.

```
ZmADF5-1    .............................MAMAYKMATEG     11
ZmADF5-2    MRDRIRRQRIPRFRRQGVHPIRDDPQATAMAMAYKMATEG     40

ZmADF5-1    MNVKEECQRWFMEMKWKKVHRFVVYKIDERSRAVLVDKVG     51
ZmADF5-2    MDVKEECQRWFMEMKWKKVHRFVVYRIDERSRAVLVDRVG     80

ZmADF5-1    GPGEGYEELVAALPGDDCRYAVFDFDFVTVDNCQKSKIFF     91
ZmADF5-2    GPGEGYEELVAALPGDDCRYAVFDFDFVSVDNCQKSKIFF    120

ZmADF5-1    IAWSPAASRIRAKILYATSKQGLRRLLDGVHYEVQATDPS    131
ZmADF5-2    IAWSPAASRIRAKILYATSKQGLRRLLDGVHYEVQATDPS    160

ZmADF5-1    EMGFDVIRGRA                                 142
ZmADF5-2    EMGFDVIRGRA                                 171
```

Supplementary Figure S2 Amino acid sequence alignment of two *ZmADF5* genes.

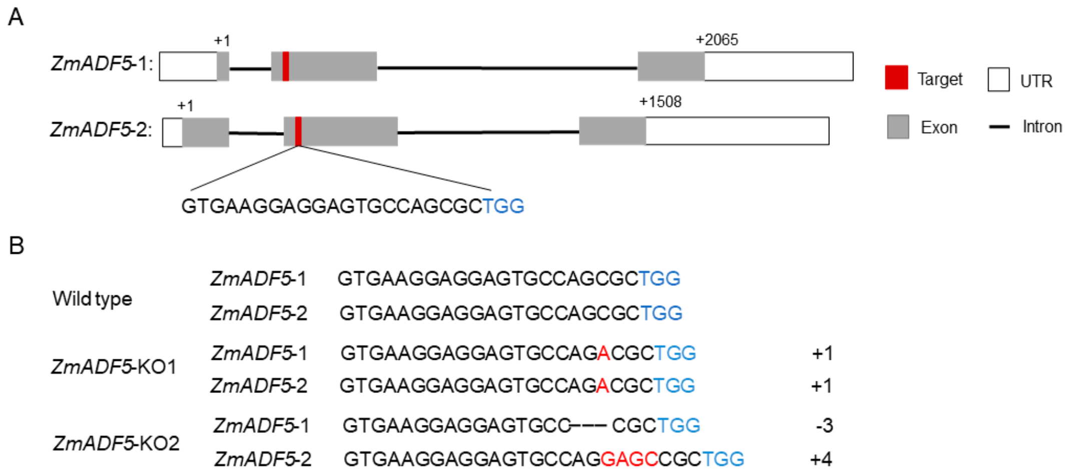

Supplementary Figure S3 Target site design of *ZmADF5* gene knockout and PCR identification of the knockout lines. **(A)** Design of the target sites of *ZmADF5*-1 and *ZmADF5*-2 genes by CRISPR/Cas9. **(B)** Insertion/deletion mutations at the target sites in the *ZmADF5* knockout lines. Black font for target sequence, blue font for PAM sequence, red base for an inserted base, dash for missing base.

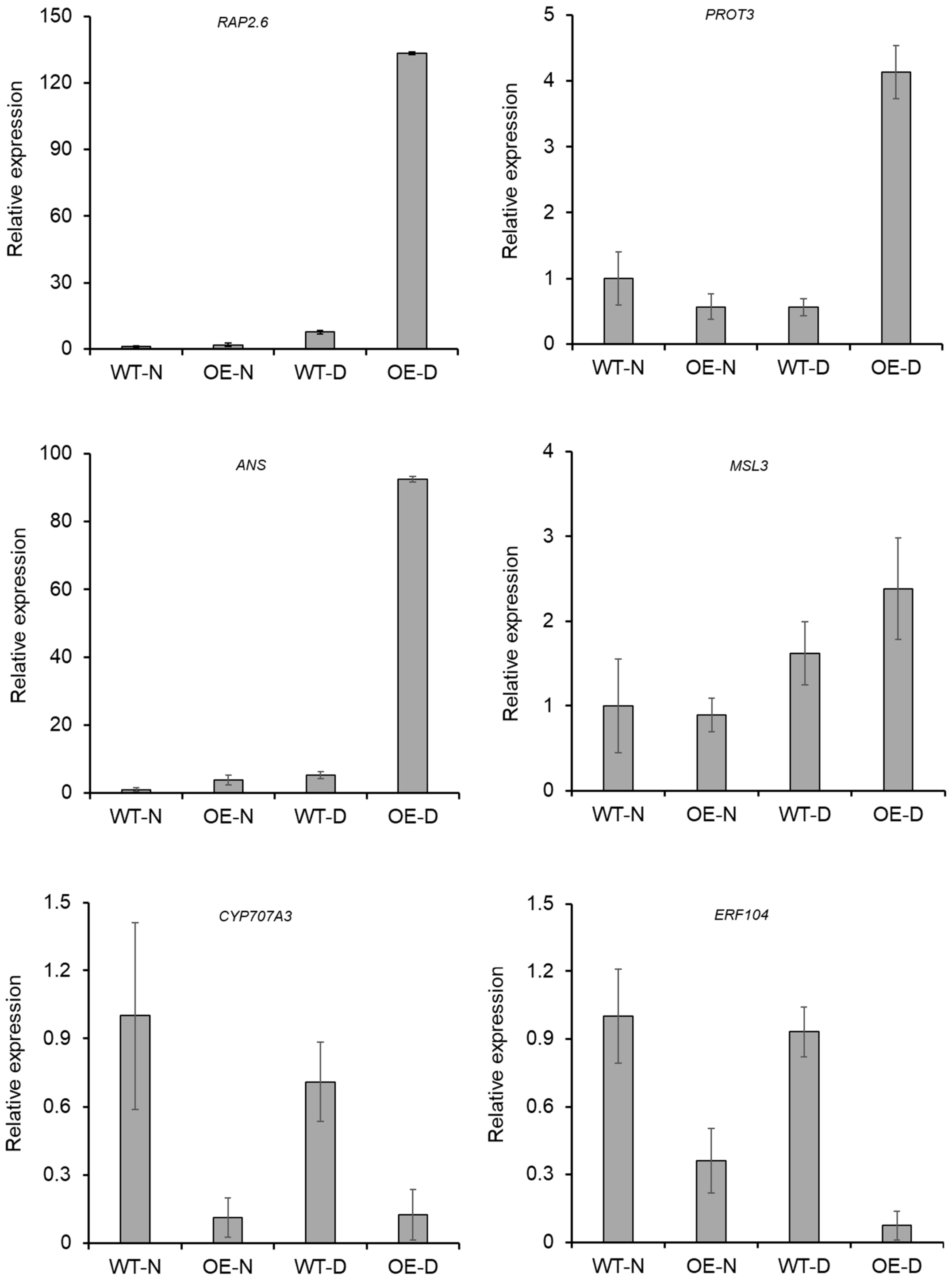

Supplementary Figure S4 Expression levels of differentially expressed genes in different plants under normal and drought conditions.

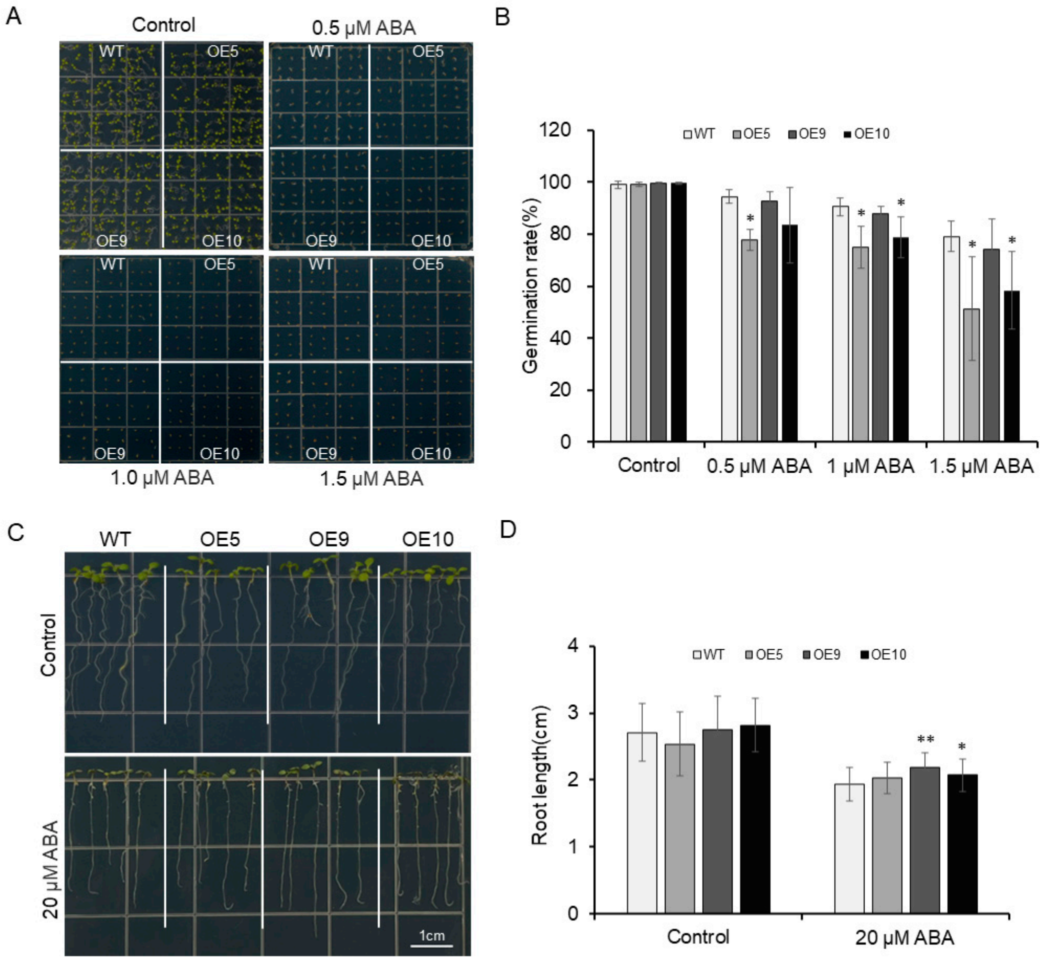

Supplementary Figure S5 ABA sensitivity of 35S:*ZmADF5* transgenic Arabidopsis plants during germination and post-germination growth. **(A)** Growth of WT and 35S:*ZmADF5* seedlings on 1/2 MS medium with or without (control) 0.5 μM ABA, 1.0 μM ABA or 1.5 μM ABA. The photographs show representative seedlings. **(B)** Statistics of germination rate of WT and 35S:*ZmADF5* transgenic Arabidopsis. **(C)** Effects of ABA on the seedling growth of WT and 35S:*ZmADF5* transgenic Arabidopsis plants on 1/2 MS medium containing 20 μM ABA. Scale bar, 1 cm. **(D)** Root length statistics of WT and 35S:*ZmADF5* transgenic plants on 1/2 MS medium containing 20 μM ABA. Significant differences were determined by t-tests. $*P < 0.05$, $**P < 0.01$.
