# Supplementary material for: ZmADF5, a Maize Actin-Depolymerizing Factor Conferring Enhanced Drought Tolerance in Maize
Source: Plants (Basel). 2024 Feb 24;13(5):619. doi: 10.3390/plants13050619 (PMC10935123; doi:10.3390/plants13050619)
Supplement: Supplementary file 1 [file plants-13-00619-s001.zip › Supplementary tables.pdf]

Supplementary Table S1. Information regarding all 78 ADF genes in maize and six other plant species

Organism	Serial number	Gene name	Gene ID	Accession number	Chromosome	Location v4
Zea mays	1	ZmADF3-2	542445	GRMZM2G060702	1	298705823..298708006
	2	ZmADFn-1	100285480	GRMZM2G064875	1	81672408..81675503
	3	ZmADF3-1	103644252	Zm00001d034643	1	298703861..298705313
	4	ZmADF2-1	103645671	GRMZM2G108807	1	289262168..289336459
	5	ZmADF5-1	100381350	GRMZM2G077942	1	33147666..33150364
	6	ZmADF2-2	542584	GRMZM2G097122	2	205994782..205995797
	7	ZmADFn-3	100285352	GRMZM2G071327	2	20017525..20018783
	8	ZmADFn-2	100193829	GRMZM2G071253	2	20013226..20016905
	9	ZmADFn-4	100282518	GRMZM2G463471	4	156845488..156847462
	10	ZmADF3-3	100280568	GRMZM2G002825	5	2443271..2445180
	11	ZmADF6	100281968	GRMZM2G130678	5	5373242..5376437
	12	ZmADFn-5	100282060	GRMZM2G015127	5	198490211..198492974
	13	ZmADF4	100192897	GRMZM2G037140	6	37509082..37510152
	14	ZmADF1	542430	GRMZM2G117603	7	154119509..154120538
	15	ZmADF5-2	100280108	GRMZM2G147775	9	145900593..145902671
Sorghum bicolor	1	ADF5	8054356	SORBI_3001G439700	1	71771097..71778096
	2	ADF10	8063189	SORBI_3001G322400	1	60971096..60972981
	3	ADF2	8084159	SORBI_3001G070000	1	5311254..5314090
	4	ADF3-2	8057046	SORBI_3001G034900	1	2635373..2636325
	5	ADF3-1	110431716	SORBI_3001G034700	1	2632418..2634799
	6	ADF7-1	8081480	SORBI_3001G417600	1	69867933..69869832
	7	ADF1-1	8080684	SORBI_3002G303500	2	67939285..67940524
	8	ADF1-2	8076067	SORBI_3004G290500	4	63125568..63127632
	9	ADF6	8055977	SORBI_3006G173400	6	52942755..52947691

	10	ADF7-2	8068689	SORBI_3009G015700	9	1474863..1477081
Arabidopsis thaliana	1	ADF11	839281	AT1G01750	1	275188..276310
	2	ADF5	816171	AT2G16700	2	7244500..7246069
	3	ADF6	817676	AT2G31200	2	13293953..13295410
	4	ADF1	823744	AT3G46010	3	16909217..16910845
	5	ADF2	823743	AT3G46000	3	16907334..16909119
	6	ADF7	828664	AT4G25590	4	13058936..13060250
	7	ADF9	829649	AT4G34970	4	16653704..16654955
	8	ADF8	828037	AT4G00680	4	279596..281072
	9	ADF3	836110	AT5G59880	5	24120164..24121949
	10	ADF4	836111	AT5G59890	5	24122348..24123912
	11	ADF10	835312	AT5G52360	5	21257934..21259408
Glycine max	1	ADF7-1	100795241	GLYMA_01G218900	1	54866040..54868160
	2	ADF6	100819975	GLYMA_03G162900	3	37777445..37779668
	3	ADF5-1	100793035	LOC100793035	4	371128..371982
	4	ADF7-2	100779214	GLYMA_05G206500	5	38926889..38929620
	5	ADF5-2	100785493	GLYMA_06G003900	6	363520..364578
	6	ADF7-3	100790487	GLYMA_08G013400	8	1051083..1052993
	7	ADF2	100813011	GLYMA_09G019200	9	1510124..1512174
	8	ADF7-4	100785437	GLYMA_11G024500	11	1745509..1747339
	9	ADF9	100801857	GLYMA_11G106600	11	8127320..8128434
	10	ADF1	100305514	GLYMA_15G125300	15	9943757..9945795
Solanum tuberosum	1	ADFn-2	102599827	LOC102599827	unknown	1126680..1129510
	2	ADF1-2	102596934	LOC102596934	unknown	1457226..1460021
	3	ADF2-1	102594782	LOC102594782	unknown	17536..18062
	4	ADF2-3	102590986	LOC102590986	unknown	178712..181291

	5	ADF12-2	102586653	LOC102586653	unknown	1939875..1941414
	6	ADF7	102597057	LOC102597057	unknown	235200..236192
	7	ADF10	102606033	LOC102606033	unknown	240916..243061
	8	ADF12-1	102593491	LOC102593491	unknown	259862..261304
	9	ADF2-2	102598836	LOC102598836	unknown	46189..48980
	10	ADF5	102593190	LOC102593190	unknown	515119..516386
	11	ADFn-1	102588600	LOC102588600	unknown	611032..614519
	12	ADF1-1	102585767	LOC102585767	unknown	958002..961423
Oryza sativa Japonica Group	1	ADF1	4330233	Os02g0663800	2	26919898..26922502
	2	ADF5	4332220	Os03g0243100	3	7565326..7567806
	3	ADF4	4334595	Os03g0820600	3	34431651..34434298
	4	ADF2	4334307	Os03g0780400	3	32360704..32363839
	5	ADF3	4334594	Os03g0820500	3	34430396..34431383
	6	ADF6	4336623	Os04g0555700	4	27819097..27823259
	7	ADF7	4337605	Os05g0113400	5	716229..718801
	8	ADF9	4343240	Os07g0484200	7	17740678..17742313
	9	ADF8	107277944	Os07g0297500	7	11644299..11646136
	10	ADF10	4349142	Os10g0521100	10	20161035..20162733
	11	ADF11	4352848	Os12g0628100	12	26888763..26890275
Setaria italica	1	ADF3-1	101766069	SETIT_037963mg	IX	1870360..1872230
	2	ADF3-2	101766473	SETIT_039957mg	IX	1872970..1873837
	3	ADF1	101763828	SETIT_019388mg	I	33723205..33725149
	4	ADF2-1	101781863	SETIT_037933mg	IX	3994768..3997798
	5	ADF7-1	101765915	SETIT_023591mg	III	4012575..4014626
	6	ADF2-2	101765611	SETIT_033222mg	II	40615294..40616310
	7	ADF10	101776880	SETIT_038556mg	IX	41037419..41043163

8	ADF7-2	101769205	SETIT_037959mg	IX	49839978..49841232
9	ADF5	101777854	SETIT_037893mg	IX	51467843..51470314

Supplementary Table S2. Primers used in this study

Gene	Forward primer (5′-3′)	Reverse primer (5′-3′)
ZmADF5-1	TGCTCAGTAGTCAGTAGTC	ACCCATCAACATCAACAC
ZmADF5-2	CGGTTCTTCCGAGAGATGGT	CCATAACAACTCTGGTCGGAAT
AtRAP2.6	GTCTCTGGTGAGACCGAACC	GGCCTCTCCAAGGACATTGA
AtANS	ATGAACGACGTTTGGGGGAA	ATCAACGAGTGGGTCACACC
AtPROT3	GAGGCGGGTACATAGCAGTT	TTGAGCCAATGGCATAGCTT
AtMSL3	TCGTACGGAGTAGGTTAATTGTGT	TGAAAGACCAGTCCGAGTCAG
AtCYP707A3	AGGGGTGTTT TGGTAATTTGCAG	TCGTTCCAAGGCAATAGGCA
AtERF104	GCGGCTAGGGTTAAAGTGGA	TACCATCTCCTGCTCCCACA
TUB4	GAGGGAGCCATTGACAACATCTT	GCGAACAGTTCACAGCTATGTTCA
GAPDH	CATCACCACGGACTACAT	GACTCCACGACATACTCA
PAN580-ZmADF5	ACCGGTCCCGGGGGATCC<br>ATGGCGATGGCGTACAAG	CTTGCTCACCATGGATCC<br>TTGCGCGCGGCCTCTGGA

Supplementary Table S3. Transgenic maize used in this study

Name	Species	Origin	Generation	Control
ZmADF5-OE1	*Zea mays* L.	*ZmADF5*-H002	BC_3F1	Zheng58
ZmADF5-OE2	*Zea mays* L.	*ZmADF5*-H005	BC_3F1	Zheng58
ZmADF5-KO1	*Zea mays* L.	EA06	T_2	C01
ZmADF5-KO2	*Zea mays* L.	EA21	T_2	C01
